# Musical Electroacupuncture May Be a Better Choice than Electroacupuncture in a Mouse Model of Alzheimer's Disease

**DOI:** 10.1155/2016/3131586

**Published:** 2016-11-16

**Authors:** Jing Jiang, Gang Liu, Suhua Shi, Zhigang Li

**Affiliations:** ^1^Beijing University of Chinese Medicine, Beijing 100029, China; ^2^Community Health Service Center of Dongcheng District, Beijing 100010, China; ^3^Third Affiliated Hospital of Beijing University of Chinese Medicine, Beijing 100029, China

## Abstract

*Objectives*. To compare musical electroacupuncture and electroacupuncture in a mouse model of Alzheimer's disease.* Methods*. In this study, 7.5-month-old male senescence-accelerated mouse prone 8 (SAMP8) mice were used as an Alzheimer's disease animal model. In the normal control paradigm, 7.5-month-old male SAMR1 mice were used as the blank control group (N group). After 15 days of treatment, using Morris water maze test, micro-PET, and immunohistochemistry, the differences among the musical electroacupuncture (MEA), electroacupuncture (EA), Alzheimer's disease (AD), and normal (N) groups were assessed.* Results*. The Morris water maze test, micro-PET, and immunohistochemistry revealed that MEA and EA therapies could improve spatial learning and memory ability, glucose metabolism level in the brain, and A*β* amyloid content in the frontal lobe, compared with the AD group (*P* < 0.05). Moreover, MEA therapy performed better than EA treatment in decreasing amyloid-beta levels in the frontal lobe of mice with AD.* Conclusion*. MEA therapy may be superior to EA in treating Alzheimer's disease as demonstrated in SAMP8 mice.

## 1. Introduction

Alzheimer's disease (AD) is a central nervous degenerative disease with memory impairment, aphasia, agnosia, and executive dysfunction, as well as personality and behavior changes [1]. Pathological features of AD include *β* amyloid (A*β*) deposition and neurofibrillary tangles, leading to progressive neuronal damage, and ultimately atrophy of the cortex and subcortical structures [2]. Magnetic resonance imaging (MRI) studies demonstrated that the hippocampus is a region affected early in AD patients [3]. However, relatively few studies are available assessing changes in other brain regions in AD patients, especially the frontal lobe. Interestingly, evaluating 41 patients with AD, Harwood et al. found that the insight and cognitive impairment as well as functional deficits in AD are associated with the glucose metabolic rate in the frontal cortex [4].

We previously demonstrated that treatment with electroacupuncture could effectively improve the spatial learning and memory ability as well as glucose metabolism in the hippocampus of animals with AD [5]. Other studies suggested that music therapy could contribute to a supplementary treatment of AD [6]. In China, an innovative therapy combined electroacupuncture and music therapy, and the term musical electroacupuncture (MEA) was coined. MEA has been clinically used to treat some neurological and psychotic disorders. Addition of musical therapy helps overcome acupuncture intolerability; therefore, the MEA therapy in a way is superior to traditional electroacupuncture [7].

In the current study, we aimed to address two questions: (1) what happens in the frontal lobe during MEA? Do these changes differ from those observed in other brain regions? (2) Are there differences between the two therapies for AD treatment? To this end, senescence-accelerated mouse prone 8 (SAMP8) mice were selected as an AD animal model, and the differences between techniques were assessed. First, Morris' water maze test was used to evaluate behavioral changes in the model animals. Then, micro-PET assessment of a region of interest (ROI) and glucose metabolism evaluation in different brain regions were performed. Finally, immunohistochemistry (IHC) was used to assess the changes of amyloid-*β*1-42 deposition in the frontal lobe after the treatments.

## 2. Materials and Methods

### 2.1. Animals

Senescence-accelerated mouse prone 8 (SAMP8) and cognate normal senescence-accelerated mouse-R1 (SAMR1) breeding pairs were kindly provided by Professor Takeda at Kyoto University, Japan [8]. All animals were male and specific pathogen-free (SPF), weighing 30 ± 2 g. They were housed in a barrier facility at the Experimental Animal Centre of First Teaching Hospital of Beijing University of Traditional Chinese Medicine, under controlled temperature (24 ± 2°C) and 12 h/12 h dark-light cycle, with sterile drinking water and standard pellet diet* ad libitum*. All experiments were performed according to the National Institute of Health Guide for the Care and Use of Laboratory Animals (NIH publications number 80-23). Thirty 7.5-month-old male SAMP8 mice were divided into three groups (*n* = 10 per group), including SAMP8 Alzheimer's disease control (AD), electroacupuncture (EA), and musical electroacupuncture (MEA) groups. Ten 7.5-month-old male SAMR1 mice composed the normal control (N) group.

### 2.2. Acupuncture

Electroacupuncture and musical electroacupuncture treatments were performed 20 minutes per day, once daily for 15 days (no treatment on day 8). Prescription of acupuncture points included DU20* Baihui*, DU 26* Shuigou*, and EX-HN3* Yintang* (significant extra points); the locations of these points were according to the National Acupuncture Society for Experimental Research developed by the “laboratory animal acupuncture atlas.”* Huatuo* card 30# (0.5 inch) needles were used for treatment. The pricking method was used for DU 26* Shuigou* and the flat thorn method for DU20* Baihui* and EX-HN3* Yintang*. Needle depth was 0.5 cm and taped.

In the EA group, the needle handle was connected to the HANS-LH202 electroacupuncture device (Peking University Institute of Science Nerve and Beijing Hua Wei Industrial Development Company), with sparse wave at 2 Hz, 2 V, and 0.6 mA.

In the MEA group, the needle handle was connected to the ZJ-12H musical electroacupuncture device (Developed by Chinese Acupuncture Society and manufactured by Harbin Zhihou Medical Devices Co., Ltd.). Clear rhythm, moderate speed, and music prescription intensity (*curing dementia prescription*) were selected; music intensity was adjusted so that the animals remained quiet during the treatment.

In the N and AD groups, no treatment was carried out, with grabbing and fixing the mice in order to ensure the same treatment conditions, once daily for 15 days.

### 2.3. Morris' Water Maze Test

Morris' water maze consisted of a circular tank (diameter, 90 cm; height, 50 cm), filled with water to a depth of 29 cm, maintained at 24 ± 1°C, and rendered opaque with blue-black ink. A removable circular platform (diameter, 9.5 cm; height, 28 cm) with the top surface 1 cm below the water was located inside the pool. The pool area was conceptually divided into four quadrants (NE, NW, SW, and SE) of equal size. Data were collected by a video camera (TOTA-450d, Japan), which was fixed to the ceiling and connected to a video recorder with an automated tracking system (China Daheng Group, Beijing, China).

In the behavioral test, mice were placed in the pool of water containing a platform just below the surface of the water. They escaped from the maze once they find the platform. Distal visual cues are arrayed around the room, and, in general, mice are able to find the location of the hidden platform based on these cues.

#### 2.3.1. Hidden Platform Test

This test assesses the ability of mice to find the platform under conditions where they cannot directly see the latter but must either remember it based on external cues or perform a search. The platform was placed 1 cm under the water surface; the water was rendered opaque by a suspension of dark blue, nontoxic tempera paint. The platform was placed in a different location from that used in the visible platform testing. Each mouse was released from one of the 4 locations and had 60 s to search for the hidden platform. At the end of each trial, the mouse was placed on the platform or allowed to stay there for 15 s. Prominent spatial cues were arrayed around the room. The investigator also constituted a powerful spatial cue and always sat in the same location during each trial after releasing the mouse. Six trials per day for 5 consecutive days were performed with the platform location kept constant. The time that the mouse took to find the platform was recorded and represented escape latency.

#### 2.3.2. Probe Trial

The day after completion of the hidden platform test, the platform was removed; each mouse was placed in the pool once for 60 s, starting from the same starting location used first in the hidden platform test. The time spent swimming in the quadrant that contained the platform was recorded. This is considered the most specific test for spatial memory. The time spent in the platform quadrant was recorded, and the percentage of total time spent swimming to the platform quadrant was derived.

### 2.4. Micropositron Emission Tomography

Four animals were randomly selected from each group for micro-PET detection. The ^18^F-FDG PET tracer was provided by the Chinese Medicine Research Institute PET Room; PET imaging was carried out on a Siemens INVEON PET/CT imaging system. Before the experiments, mice (7.5 months, 28~32 g) were submitted to blood glucose monitoring and showed levels in the normal range (7.0~10.1 mmol/L). Therefore, they could be assessed by micro-PET. Mice were deprived of water 6 h before assessment. The animals were placed in the suction chamber, inhaling oxygen mixed with 1.5% isoflurane for anesthesia. After complete anesthesia, approximately 14.8~16.5 MBq^18^F-FDG PET were injected via the tail vein. After ^18^F-FDG PET tracer uptake for 60 min, the mice were placed in the prone position, parallel to the scanner long axis, with the head located within the scanner field of view. Then, micropositron emission tomography began to collect images. The mice were anesthetized by inhalation of oxygen mixed with 1.5% isoflurane (1 L/min).

#### 2.4.1. Micropositron Emission Tomography Image Reconstruction

Filtered back projection (FBP) and CT photon attenuation correction were used for image reconstruction. Dynamic micro-PET image frames were taken at 30 s/frames.

#### 2.4.2. Region of Interest Selection

Three-dimensional regions of interest were selected in the hippocampus, in transverse, coronal, and sagittal planes. The uptake rate per gram in each region of interest was calculated.

### 2.5. Immunohistochemistry

After the Morris water maze test, the remaining six mice in each group (four were used in micro-PET) were anesthetized by intraperitoneal injection of 10% chloral hydrate at 0.35 mL/100 g body weight. Three minutes later, the chest was opened and the heart exposed; intubation was performed from the left ventricle to the ascending aorta with quick injection of 100 mL saline. Then, the right atrial appendage was cut, and 4% paraformaldehyde was injected until the liver turned white with clear fluid flowing out from the right atrial appendage. After the perfusion, the mouse was decapitated and the whole brain extracted and placed on ice. The brain was then placed into 4% paraformaldehyde for paraffin embedding.

For histochemistry, paraffin embedded brain tissue sections were deparaffinized with xylene and hydrated with graded alcohol. Then, the sections were treated with citric acid antigen repair buffer and washed with PBS (pH 7.4) every 5 min three times with shaking. After incubation with 3% hydrogen peroxide for 20 min in the dark to quench endogenous peroxidase, the sections were incubated with anti-A*β*1-42 antibody (1 : 50, ab10148, Abcam) overnight. Then, secondary antibodies were added for 30 min at room temperature, and detection was performed with DAB. Finally, the sections were dehydrated with graded alcohol and mounted. Micrographs of brain tissue samples were obtained at 400x magnification, and integral optical density (IOD) values were calculated using Image-Pro Plus 6.0 software.

### 2.6. Statistical Analysis

Data are mean ± SD for each group. For the Morris water maze test, the escape latency time of the hidden platform trial was analyzed by the Huynh-Feldt test, while one-way ANOVA was conducted on probe trial, micro-PET test, and immunohistochemical data. LSD test was used to compare group pairs. Statistical significance was set at *P* < 0.05. All statistical analyses were performed with the SPSS software V.17.0 (SPSS, USA).

## 3. Results

### 3.1. Effects of Electroacupuncture and Musical Electroacupuncture on Spatial Learning and Memory Ability of SAMP8 Mouse in Morris' Water Maze Test

**Figure 1:**
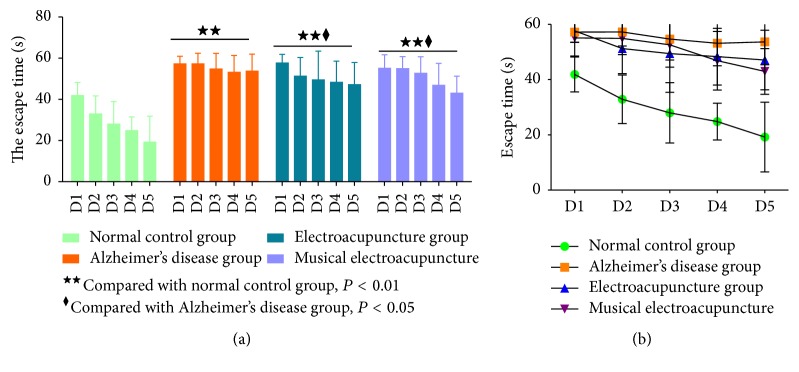


The effects of electroacupuncture and musical electroacupuncture in spatial location ability of SAMP8 mice in the WMW test are shown in Figure 1(a). With training time extension, escape latency in all groups showed a downward trend (Figure 1(b)). The AD group showed a marked retardation in escape latency compared with the N group (*P* = 0.00), probably due to memory deficits resulting from the rapid aging process impairing learning and memory. Compared with the AD group, escape latency in the EA (*P* = 0.031) and MEA (*P* = 0.023) groups was significantly reduced (*P* < 0.05). As shown in Figures 1(a) and 1(b), the MEA group performed better than the EA group, although there was no statistically significant difference (*P* = 0.895).

**Figure 2:**
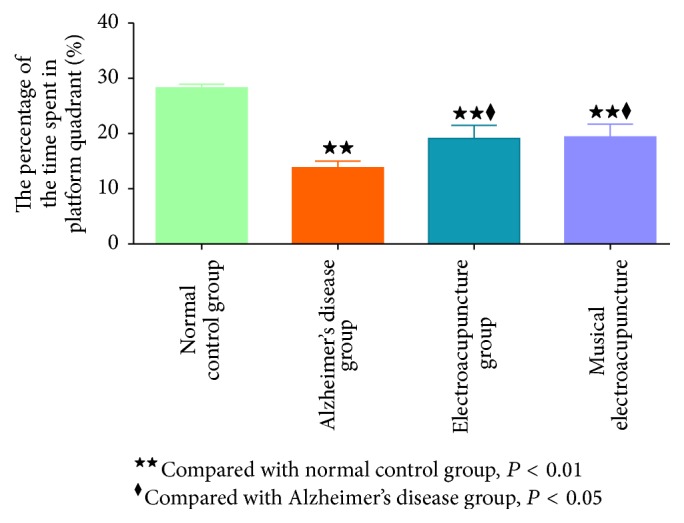


To assess therapeutic effects on spatial memory ability, performance on day 6 was examined by analyzing the percentages of swimming time in the expected platform position. A higher percentage of time spent in the platform quadrant is interpreted as a higher level of memory retention [9]. In this trial, compared with the AD group, the EA (*P* = 0.045) and MEA (*P* = 0.035) groups showed increased time spent in the platform quadrant (*P* < 0.05). What is more, percentages of time spent in the platform quadrant were similar between the EA and MEA groups (*P* = 0.907, Figure 2).

Besides the escape time, the average animal swimming speed is also associated with spatial learning and memory ability. In some cases, swimming speed in the normal control group was slower, with the escape time longer than in the Alzheimer's disease group. Thus, escapes times cannot serve as the only indicator of learning ability and memory. The average swimming speed before getting on the platform is an important indicator of exercise ability and can reflect individual differences in experimental animals.

**Figure 3:**
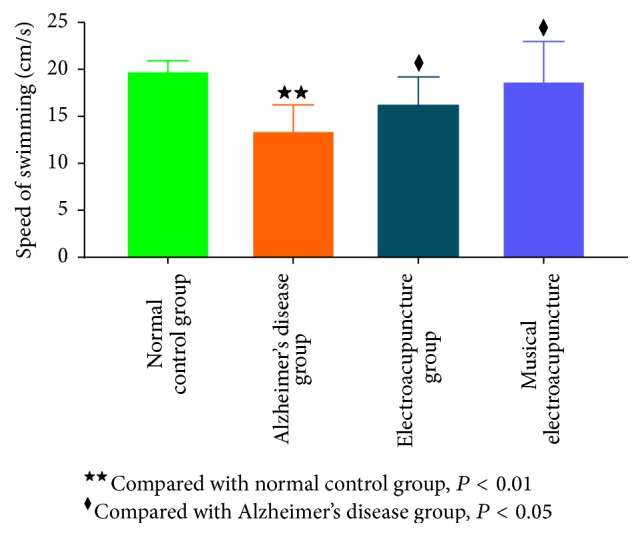


Compared with the N group, the swimming speed in AD group was significantly reduced (*P* = 0.00), indicating lower exercise ability for SAMP8 mice compared with SAMR1 animals. Compared with the AD group, electroacupuncture and musical electroacupuncture both increased the swimming speed of SAMP8 mice (EA, *P* = 0.023; MEA, *P* = 0.009). What is more, the MEA group performed better than the EA group (Figure 3).

### 3.2. PET Images and ^18^F-FDG Uptake Rate in Different Brain Regions

**Figure 4:**
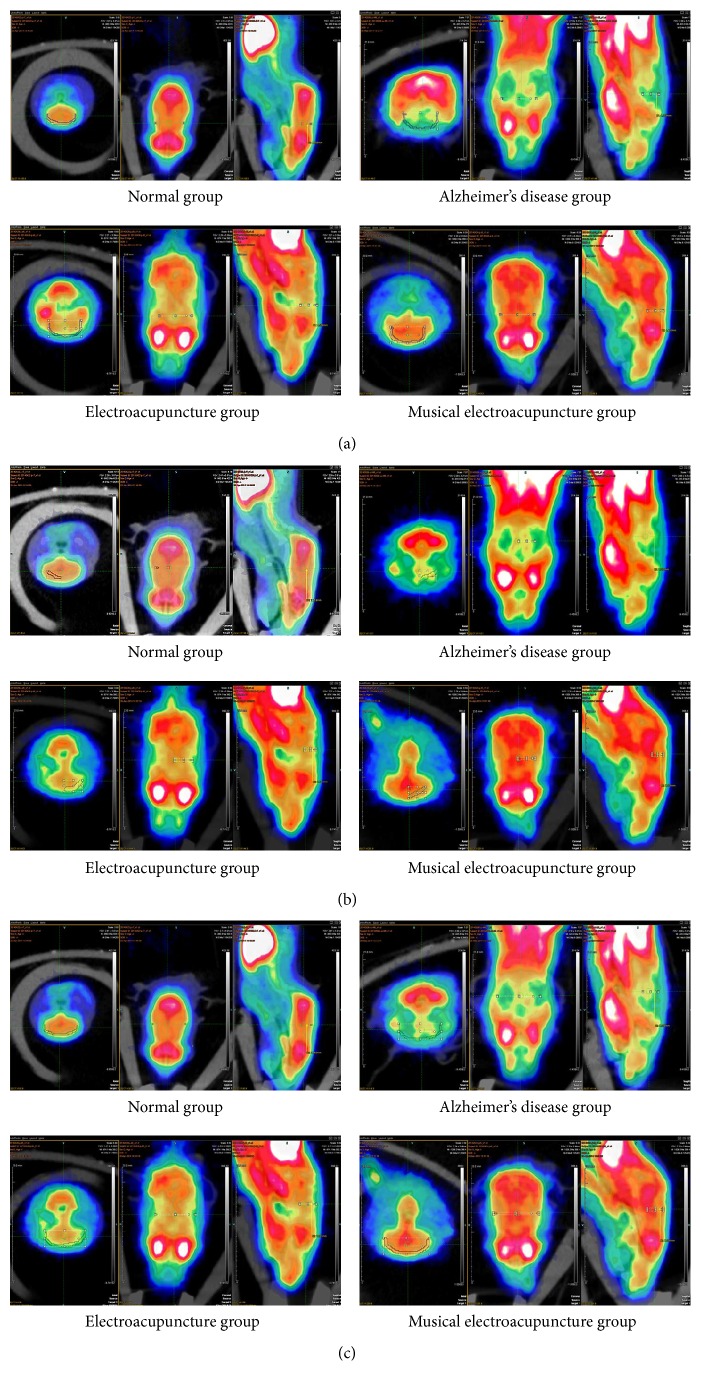
(a) The micro-PET images of the frontal lobe. (b) The micro-PET images of the hippocampus. (c) The micro-PET images of the cerebral cortex.

Micro-PET images were obtained from the hippocampus, frontal lobe, and cerebral cortex of each mouse. The same color standard and code were used from top to bottom to display the metabolic rate of glucose. After treatment with electroacupuncture and musical electroacupuncture, ^18^F-FDG levels in each brain region assessed were higher than values obtained for the nontherapy group (Figures 4(a), 4(b), and 4(c)).

**Figure 5:**
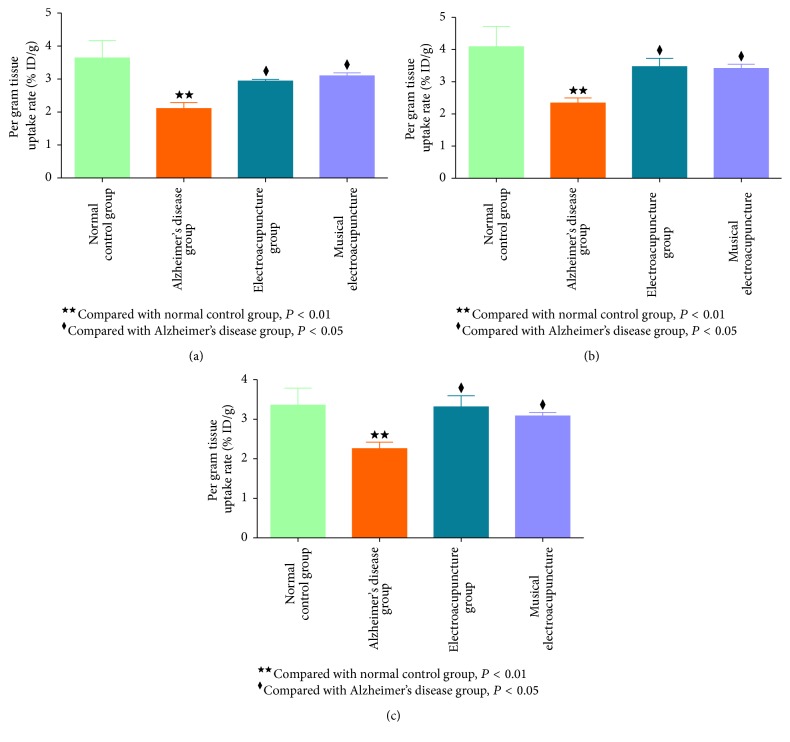
(a) The uptake rate of ^18^F-FDG per gram in the frontal lobe of each group. (b) The uptake rate of ^18^F-FDG per gram in the hippocampus of each group. (c) The uptake rate of ^18^F-FDG per gram in the cerebral cortex of each group.

To obtain acute differences in glucose metabolism among groups, the uptake rate of ^18^F-FDG per gram of different brain regions was assessed. After treatment with electroacupuncture and musical electroacupuncture, uptake rates of ^18^F-FDG per gram in the hippocampus (EA, *P* = 0.039; MEA, *P* = 048), cerebral cortex (EA, *P* = 0.14; MEA, *P* = 0.047), and frontal lobe (EA, *P* = 0.045; MEA, *P* = 0.031) were higher than those obtained for the Alzheimer's disease group (Figures 5(a), 5(b), and 5(c)). In the frontal lobe, the uptake rate of ^18^F-FDG for the MEA group was higher than that of the EA group, while, in the other two regions, the EA group showed higher values.

### 3.3. Protein Expression of Amyloid-*β*1-42 in the Frontal Lobe

**Figure 6:**
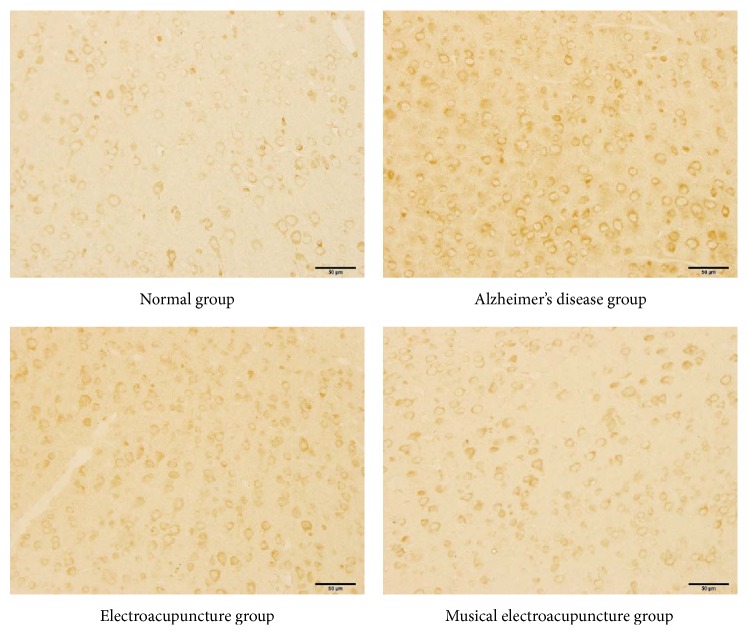
The IHC images of frontal lobe.

**Figure 7:**
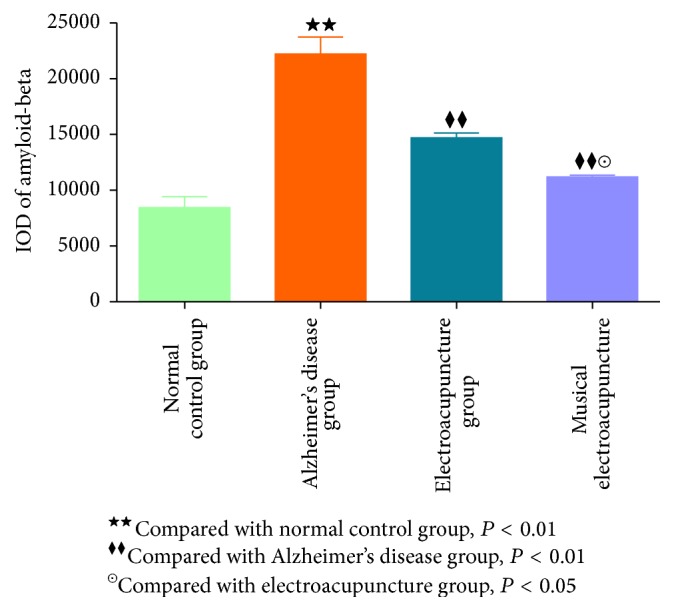
The IOD of amyloid-beta in frontal lobe of each group.

After the behavioral tests and imaging, brain tissue samples were analyzed by immunohistochemistry to assess the effects of the two therapeutic variants on amyloid-*β*1-42 accumulation due to neuronal damage and memory impairment. Compared with the N group, IOD of amyloid-*β*1-42 in the frontal lobe was significantly higher in the AD group (*P* = 0.00), EA (*P* = 0.00), and MEA (*P* = 0.007) groups. Meanwhile, IOD of amyloid-*β*1-42 in frontal lobe samples from the AD group was significantly higher than those of the EA (*P* = 0.00) and MEA (*P* = 0.00) groups. Interestingly, IOD of amyloid-*β*1-42 in frontal lobe samples from the MEA group was significantly lower than that of the EA group (*P* = 0.01 < 0.05) (Figures 6 and 7).

## 4. Discussions

### 4.1. Senescence-Accelerated Mouse Prone 8 (SAMP8) Is an Ideal Animal Model for Alzheimer's Disease

The senescence-accelerated mouse (SAM) is an accelerated aging model that was established through phenotypic selection from a common genetic pool of the AKR/J mouse strain [10]. These animals develop deficits in learning and memory relatively early in their lifespan [11]. It was shown that senescence-accelerated mouse (SAMP8), as a model of aging, displays many features known to occur in the early stage of AD such as increased oxidative stress, amyloid-beta level alteration, and tau phosphorylation [12]. What is more, published data [13] and our previous research [5] demonstrated that SAMP8 could undergo acupuncture therapy to improve learning and memory ability. In this study, therefore, SAMP8 mice were selected as an ideal animal model for Alzheimer's disease.

### 4.2. An Innovative Therapy for Alzheimer's Disease: Musical Electroacupuncture Therapy

Music therapy is a nonpharmacological treatment for the behavioral and psychological symptoms of Alzheimer's disease [14]. Appropriate music formulation could provide a form of relief to the AD patient and may stimulate cognitive activities so that areas subject to progressive degeneration are maintained [15]. In the recent ten years, increasing evidence suggests that proper music formulation could improve AD symptoms [16–21], especially for mild cases [22].

Besides, since music therapy is nonpharmacological, it is often used in combination with other therapies. Interestingly, a research on cerebral palsy combined acupuncture treatment and music therapy. Interestingly, the combined therapy showed improved outcome compared with monotherapies [23].

Therefore, in this research, we introduced musical electroacupuncture for AD treatment. Musical electro-acupuncture (MEA) combines music therapy and electro-acupuncture; during symptomatic selection of music, the sound wave could be turned into a pulse current [24]. Therefore, the effects of MEA on the human auditory organ and acupoints were separately assessed. Specifically, the fundamental characteristics and advantages included two aspects: music therapy and irregular pulse current [7].

Electroacupuncture (EA) is widely used in clinical practice and research and in experimental investigations into the mechanisms of acupuncture [25]. This therapy has been applied for AD treatment and could improve the cognitive function [26] and brain energy metabolism [5, 27]. Therefore, EA is considered an effective therapeutic intervention for AD [28]. However, the concept of* EA tolerance* had been demonstrated three decades ago [29]. The mechanism of* EA tolerance* is that the central nervous system releases analgesic substances (including brain norepinephrine and endogenous antiopioid substances) as well as large amounts of endogenous monoamine. Among them, cholecystokinin octapeptide (CCK-8) is by far the most recognized antiopioid contributing to* EA tolerance* [30], which is caused by long-term use of the same frequency in EA. Since AD treatment requires a long-term course, it likely results in* EA tolerance* [31]. Therefore, we assessed MEA in this study for AD treatment. MEA therapy was full of Chinese characteristic musical therapy. It transformed musical rhythms into constantly changing frequencies and waveforms to overcome the shortcomings of human body's tolerance of general electroacupuncture. Meanwhile, patients were allowed to listen to relieving music, which plays a role of music therapy [32]. MEA therapy is not novel in Chinese clinical and fundamental research. It was shown that such therapy performs better than traditional electroacupuncture in relieving pains [33], improving the symptoms of dermatosis (urticarial disease [34], chloasma [35]), alleviating nervous system diseases such as depression [36–38], insomnia [39], and anxiety [40, 41]. However, there was seldom research on dementia. Therefore, introducing this therapy for Alzheimer's disease constitutes an innovation.

### 4.3. Difference between MEA and EA in the Frontal Lobe of SAMP8 Mice

Studies assessing the pathogenesis of AD are currently more focused on the temporal lobe, parietal cortex, and hippocampus, with few analyzing changes in the frontal lobe [42]. Recent studies found that transgenic mice with Alzheimer's disease show early cognitive decline related to frontal atrophy, with the changes taking place even earlier than in the hippocampus [43].

As shown above, although the MEA therapy performed better than EA in the Morris water maze test, differences were not statistically significant. In micro-PET, EA therapy showed higher glucose metabolism improvement in the hippocampus and cerebral cortex compared with MEA. Only in the frontal lobe, MEA therapy was better than EA, but the difference was not significant. These findings prompted the question whether MEA therapy was more inclined to play a role in the frontal lobe.

To address this, amyloid-beta levels were assessed in the frontal lobe of SAMP8 mice, and the results confirmed the above notion. Comparing the different therapies in IOD of amyloid-beta in the frontal lobe, MEA therapy performed significantly better than EA (*P* < 0.05). Therefore, MEA indeed is inclined to play a role in the frontal lobe.

## 5. Conclusions

Using behavioral tests,* in vivo* imaging, and protein detection, the differences between MEA and EA therapy for AD treatment were assessed in SAMP8 mice. Interestingly, both EA and MEA could improve spatial learning and memory ability, improving glucose metabolism in different brain regions and amyloid-beta expression in the frontal lobe. What is more, the MEA therapy performed better than EA in decreasing amyloid-beta amounts in the frontal lobe. However, further studies are required to further explain this phenomenon.
